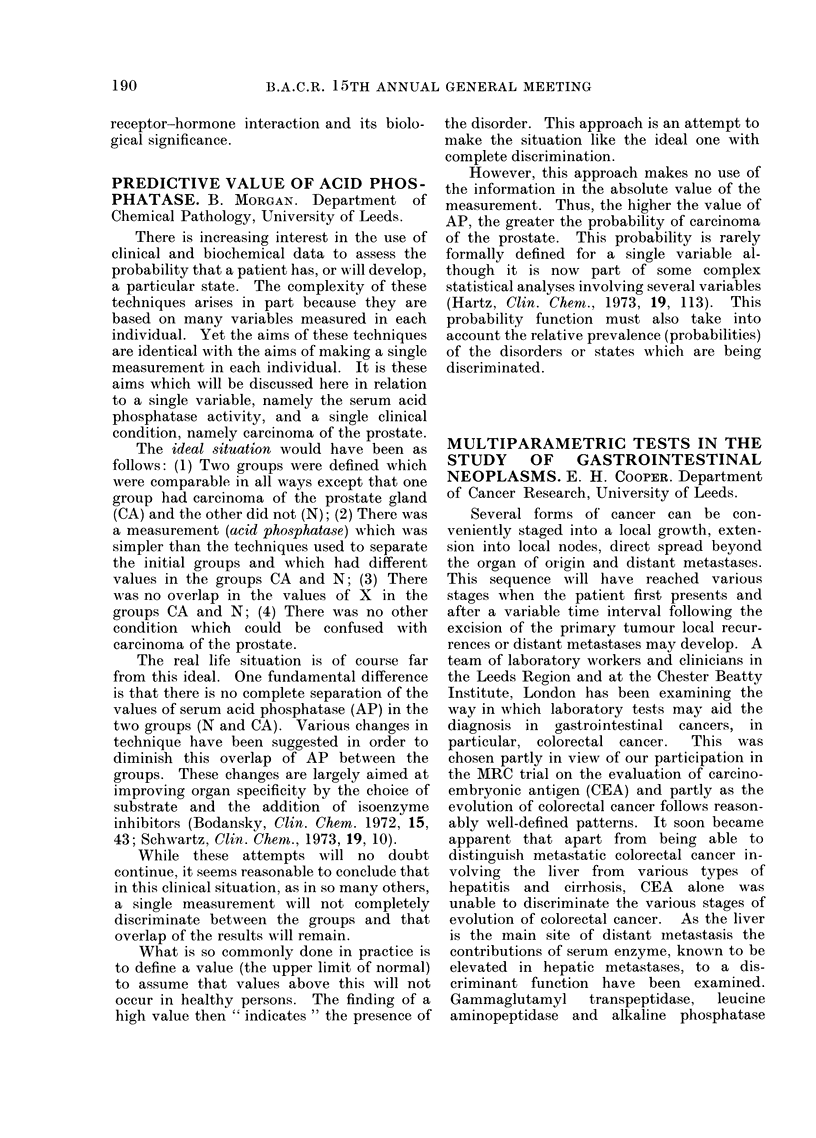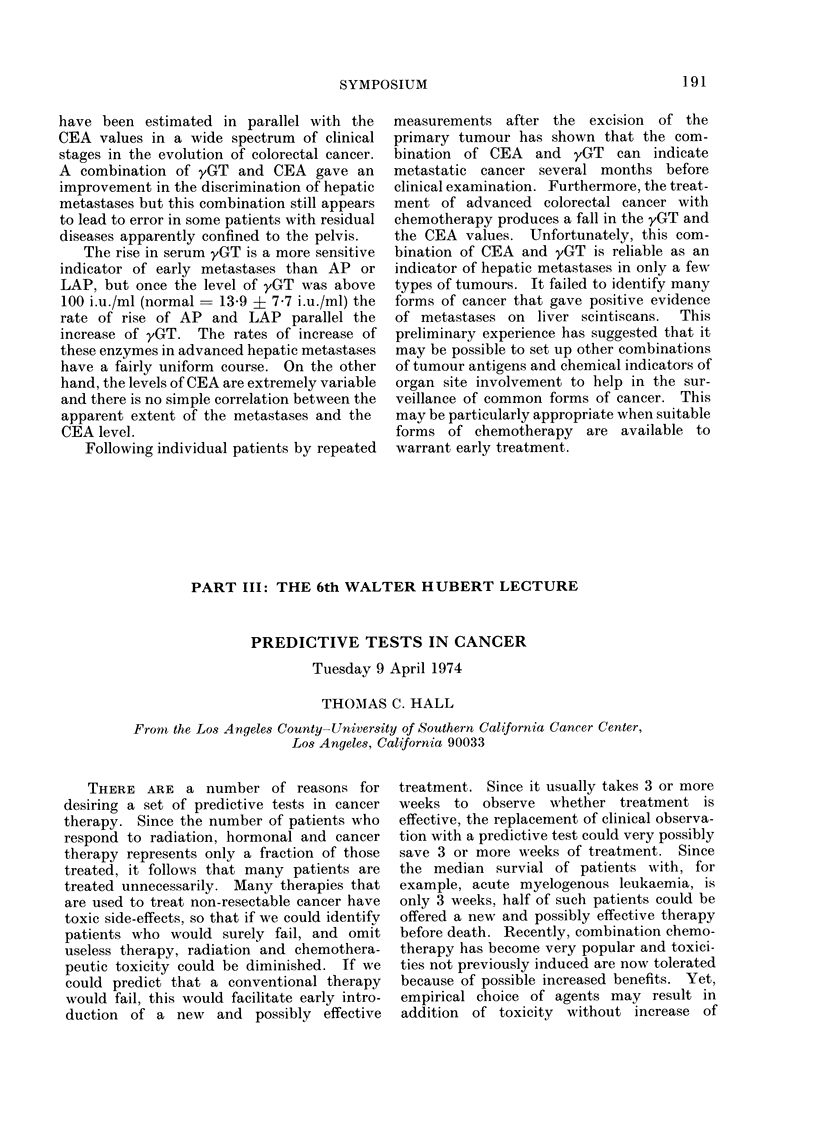# Proceedings: Multiparametric tests in the study of gastrointestinal neoplasms.

**DOI:** 10.1038/bjc.1974.180

**Published:** 1974-08

**Authors:** E. H. Cooper


**MULTIPARAMETRIC TESTS IN THE STUDY OF GASTROINTESTINAL NEOPLASMS.** E. H. COOPER. Department of Cancer Research, University of Leeds.

Several forms of cancer can be conveniently staged into a local growth, extension into local nodes, direct spread beyond the organ of origin and distant metastases. This sequence will have reached various stages when the patient first presents and after a variable time interval following the excision of the primary tumour local recurrences or distant metastases may develop. A team of laboratory workers and clinicians in the Leeds Region and at the Chester Beatty Institute, London has been examining the way in which laboratory tests may aid the diagnosis in gastrointestinal cancers, in particular, colorectal cancer. This was chosen partly in view of our participation in the MRC trial on the evaluation of carcinoembryonic antigen (CEA) and partly as the evolution of colorectal cancer follows reasonably well-defined patterns. It soon became apparent that apart from being able to distinguish metastatic colorectal cancer involving the liver from various types of hepatitis and cirrhosis, CEA alone was unable to discriminate the various stages of evolution of colorectal cancer. As the liver is the main site of distant metastasis the contributions of serum enzyme, known to be elevated in hepatic metastases, to a discriminant function have been examined. Gammaglutamyl transpeptidase, leucine aminopeptidase and alkaline phosphatase have been estimated in parallel with the CEA values in a wide spectrum of clinical stages in the evolution of colorectal cancer. A combination of $\gamma$GT and CEA gave an improvement in the discrimination of hepatic metastases but this combination still appears to lead to error in some patients with residual diseases apparently confined to the pelvis.

The rise in serum $\gamma$GT is a more sensitive indicator of early metastases than AP or LAP, but once the level of $\gamma$GT was above 100 i.u./ml (normal = $13{\cdot}9 \pm 7{\cdot}7$ i.u./ml) the rate of rise of AP and LAP parallel the increase of $\gamma$GT. The rates of increase of these enzymes in advanced hepatic metastases have a fairly uniform course. On the other hand, the levels of CEA are extremely variable and there is no simple correlation between the apparent extent of the metastases and the CEA level.

Following individual patients by repeated measurements after the excision of the primary tumour has shown that the combination of CEA and $\gamma$GT can indicate metastatic cancer several months before clinical examination. Furthermore, the treatment of advanced colorectal cancer with chemotherapy produces a fall in the $\gamma$GT and the CEA values. Unfortunately, this combination of CEA and $\gamma$GT is reliable as an indicator of hepatic metastases in only a few types of tumours. It failed to identify many forms of cancer that gave positive evidence of metastases on liver scintiscans. This preliminary experience has suggested that it may be possible to set up other combinations of tumour antigens and chemical indicators of organ site involvement to help in the surveillance of common forms of cancer. This may be particularly appropriate when suitable forms of chemotherapy are available to warrant early treatment.